# Molecular Testing of Environmental Samples as a Potential Source to Estimate Parasite Infection

**DOI:** 10.3390/tropicalmed9100226

**Published:** 2024-09-26

**Authors:** Rojelio Mejia, Barton Slatko, Cristina Almazan, Ruben Cimino, Alejandro Krolewiecki, Natalia Montellano Duran, Jacob Edwin Valera Aspetty, Paola Andrea Vargas, Chiara Cássia Oliveira Amorim, Stefan Michael Geiger, Ricardo Toshio Fujiwara, Juan David Ramirez, Luz Marina Llangarí-Arizo, Irene Guadalupe, Liliana E. Villanueva-Lizama, Julio Vladimir Cruz-Chan, María Leticia Ojeda, Eva Mereles Aranda, Sandra Ocampos Benedetti, Maritza Dalí Camones Rivera, Eddyson Montalvo Sabino, Carlos Pineda, Eric J. Wetzel, Philip J. Cooper

**Affiliations:** 1 National School of Tropical Medicine, Baylor College of Medicine, Houston, TX 77030, USA; barton.slatko@gmail.com; 2 Instituto de Investigaciones de Enfermedades Tropicales, Universidad Nacional de Salta, Salta 4400, Argentina; cristina.almazan90@gmail.com (C.A.); rubencimino@gmail.com (R.C.); alekrol@hotmail.com (A.K.); 3 Escuela de Medicina, Universidad Internacional del Ecuador UIDE, Quito 170411, Ecuador; lullangari@uide.edu.ec; 4 Laboratorio de Parasitología, Centro de Investigaciones Regionales “Dr. Hideyo Noguchi”, Universidad Autónoma de Yucatán, Mérida 97000, Mexico; liliana.villanueva@correo.uady.mx (L.E.V.-L.); vladimir.cruz@correo.uady.mx (J.V.C.-C.); 5 Centro de Investigaciones Medicas, Facultad de Ciencias de la Salud, Universidad Nacional Del Este, Minga Guazu 7420, Paraguay; letiojeda@hotmail.com (M.L.O.); evitamereles80@gmail.com (E.M.A.); socampos@med.una.py (S.O.B.); 6 Ingeniería en Biotecnología, Universidad Católica Boliviana San Pablo, Santa Cruz de la Sierra 537, Bolivia; nmontellano@ucb.edu.bo (N.M.D.); jacobvalera0410@gmail.com (J.E.V.A.); paola.a.vargas.f@gmail.com (P.A.V.); 7 Departamento de Parasitologia, Universidade Federal de Minas Gerais, Belo Horizonte 31270-901, Brazil; chiaramorim@hotmail.com (C.C.O.A.); stefan.geiger76@gmail.com (S.M.G.); rtfujiwara@gmail.com (R.T.F.); 8 Facultad de Ciencias Naturales, Universidad del Rosario, Bogotá 110141, Colombia; juand.ramirez@urosario.edu.co; 9 Molecular Microbiology Laboratory, Department of Pathology, Molecular and Cell-Based Medicine, Icahn School of Medicine at Mount Sinai, New York, NY 10029, USA; 10 IESS Hospital, Puyo 160101, Ecuador; irene82iess@gmail.com; 11 Facultad de Medicina Veterinaria y Zootecnia, Universidad Nacional Hermilio Valdizán, Huánuco 10003, Peru; marydalina15@gmail.com (M.D.C.R.); purovet2012@gmail.com (C.P.); 12 Instituto de Investigación de Enfermedades Tropicales, Universidad Nacional Toribio Rodríguez de Mendoza, Chachapoyas 01001, Peru; eddysonmont@gmail.com; 13 Department of Biology, and Global Health Initiative, Wabash College, Crawfordsville, IN 47933, USA; wetzele@wabash.edu; 14 Institute of Infection and Immunity, St George’s University of London, London SW17 0RE, UK

**Keywords:** parasites, soil, molecular testing, quantitative polymerase chain reaction, Latin America

## Abstract

We discuss the potential usefulness of molecular testing of soil, dust, and water samples to detect medically important parasites, and where such testing could be used to supplement stool sampling in humans. A wide variety of parasites including protozoa and helminths, many of which are zoonotic, have an important infection reservoir in the environment. In some cases, this environmental period is essential for further parasite development. We describe the progress in implementing methods for the molecular detection of these parasites in soil across eight collaborating centers in Latin America and represent a variety of potential applications in improving our understanding of parasite epidemiology and mapping, surveillance, and control of these parasites. This methodology offers new opportunities for improving our understanding of a wide variety of parasites of public health importance and novel tools for their control.

## 1. Introduction

Human parasitic infections acquired from the environment are estimated to infect several billion people worldwide [1]. These infections are most prevalent in children living in urban neighborhoods and rural communities in conditions of inadequate clean water and sanitation in tropical and temperate regions, especially in low- and middle-income countries (LMICs). Among these parasites are intestinal protozoa (e.g., *Giardia intestinalis* and *Cryptosporidium* spp.), soil-transmitted helminths (STH—e.g., *Ascaris lumbricoides*, *Trichuris trichiura*, hookworms, and *Strongyloides stercoralis*), and zoonotic infections (e.g., *Toxocara* spp.). In most endemic settings, individuals may be infected with more than one parasite at any time and with several parasites over their life span. Infections are generally acquired through ingesting contaminated food, water, geophagy, and other contact forms with fecal-contaminated soil. Infections with these parasites during childhood have been associated with significant mortality and morbidity, the latter primarily mediated through chronic nutritional deficits, anemia, and impaired childhood growth and development [2].

## 2. Soil as a Reservoir for Parasite Infections

In the case of protozoal cysts and helminth eggs, infectious stages of parasites in the environment can survive for extended periods in the soil, generally for months to years. For some parasites, the soil is a necessary part of their life cycles: eggs of some parasite species require a period of embryonation (e.g., *A. lumbricoides* and *T. trichiura*) before becoming infectious. In contrast, those of other species hatch into infectious larvae (e.g., hookworms) or undergo further larval development (e.g., *Strongyloides stercoralis*). Soil represents an important environmental reservoir for these infections. The long-term survival of infectious stages in the environment, whether as cysts, eggs, or larvae, depends on several factors, including soil type and characteristics (e.g., pH), climate (temperature and humidity), and shade. The presence of infectious stages in the environment, their density and dispersal, and the opportunities for transmission are likely important determinants of the endemicity of any specific parasite in any geographic locality.

## 3. Soil and Stool Sampling

Epidemiological studies of environmental parasites in endemic communities rely on stool sampling or other diagnostic methods from members of whole communities or specific groups considered to be at the greatest risk [3]. For example, STH prevalence studies often sample stools from children of school age because they are both easily accessible in schools and an important reservoir of infection. Stool sampling, particularly in large surveys, can be expensive and logistically challenging, requiring repeated visits to schools or communities, often by experienced field teams, while social and cultural factors may limit participation rates. We successfully used a multi-parallel real-time quantitative Polymerase Chain Reaction (qPCR) platform designed for use with stool samples for use with a variety of environmental samples including soil, dust, and water samples. Multiparallel qPCR is a molecular test developed to quantify the amount of parasite DNA in stool samples and is run separately for each parasite (i.e., multiparallel). This qPCR platform has now been successfully applied in multiple settings including in the USA [4,5,6], Latin America [7,8,9,10,11,12], and Africa [13,14]. Soil testing would complement stool sampling and be advantageous for the epidemiological mapping of enteric parasites, particularly those that cause significant morbidity and are amenable to control strategies. In addition, soil sampling could be used for studies of routes and intensity of transmission of a variety of parasitic pathogens, including emerging and zoonotic infections that may not be detectable in human stool samples (e.g., *Toxocara* spp. and *Angiostrongylus* spp.).

## 4. A Robust and High Throughput Molecular Assay for the Detection of Parasites in Soil, Dust, and Water

**Figure 1:**
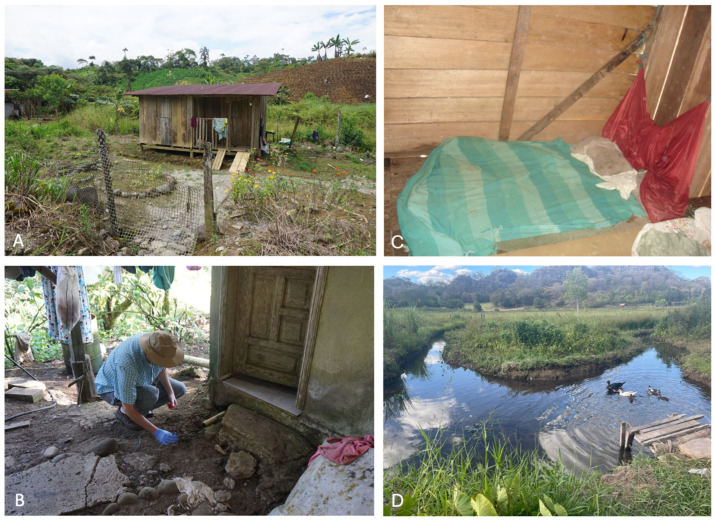
(**A**)—Soil samples were collected in the environment (i.e., in front of the front door, in the porch area, and front of the latrine) around this rural household in the Amazon region of Ecuador. (**B**)—Collection of peri-domestic soil samples in Ecuador. (**C**)—A typical bed where dust was collected in Esmeraldas Province, Ecuador. (**D**)—Local streams and waterways used for bathing and washing and which are sources of *Schistosoma mansoni* exposure in Januária, Brazil.

**Figure 2:**
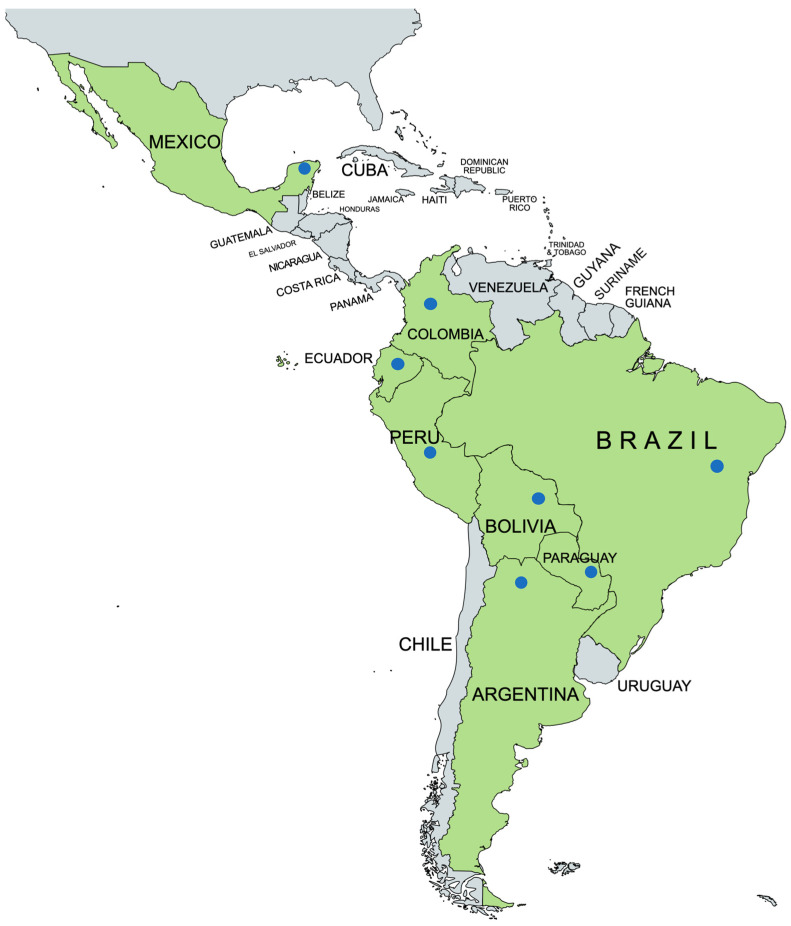
Map showing locations of collaborating centers in Latin America for our collaborating research group on using molecular methods for parasite detection in soil samples. Including sites in Argentina, Bolivia, Brazil, Colombia, Ecuador, Mexico, Paraguay, and Peru.

We evaluated the utility of qPCR-based screening for parasite infections in soil, dust, and water samples around households (Figure 1) and community centers in rural communities known to be endemic for a variety of STH and protozoan parasites in eight Latin American countries (Argentina, Bolivia, Brazil, Colombia, Ecuador, Mexico, Paraguay, and Peru) (Figure 2). Before its use in Latin America, our soil and qPCR assays were used throughout the USA. A soil study of urban parks in New York City showed a higher prevalence and parasite burden of *Toxocara* in poorer neighborhoods [15]. Another study of parasites in playground soil from indigenous Paiute reservations in Utah has similar associations of parasites with poverty [16]. Finally, a multi-state soil study in rural Southern USA (Texas, Louisiana, Mississippi, Alabama, and South Carolina) consistently revealed parasite burden and prevalence with high poverty rates [17]. Apart from considerations of climate and soil, the presence, dispersal, and density of enteric parasites in soil are likely to directly reflect the epidemiology of these parasites in communities where human populations live. We used highly specific and sensitive qPCR to map the spatial epidemiology of multiple enteric parasites within these communities. The parasites evaluated included anthroponotic and zoonotic helminths (*Ancylostoma duodenale*, *Ancylostoma ceylanicum*, *Ascaris lumbricoides*, *Necator americanus*, *Strongyloides stercoralis*, *Taenia solium*, *Trichuris trichiura*, *Toxocara canis/cati*) and protozoa (*Acanthamoeba* spp., *Blastocystis*, *Cryptosporidium* spp., *Entamoeba histolytica*, and *Giardia intestinalis*). These data have provided information on potentially important sites of transmission within households and communities for a range of different protozoal and helminth parasites, including zoonotic infections. Outdoor sampling can be extended to samples collected indoors on different surfaces, including dust. For example, we showed that mattress and floor dust samples from the beds of children living in rural communities have significant rates of parasite contamination—for example, in one analysis, 39% of mattress samples were positive for *A. lumbricoides* and positivity was strongly associated with the presence of active infections [18] (Figure 1C—a typical bed in Esmeraldas Province, Ecuador). Parasites including *G. intestinalis*, *Blastocystis*, *Cryptosporidium*, and the trematode, *Schistosoma mansoni* in Brazil, were also detected in the water samples (Figure 1D). Of course, detecting DNA by qPCR could reflect non-viable parasites rather than viable infectious parasites. Still, several methods could be used to determine the potential for transmission. Among these are the incorporation of vital dyes into living cells or the detection of mRNA [19]. Because the qPCR assays we used can detect as little as 1 fg/µL of DNA, their exquisite sensitivity must be considered, particularly if parasite viability measures are impractical, as may be the case in many settings. Under such circumstances, it may be possible to define quantitative thresholds below which it is unlikely that viable parasite stages of specific parasites are present in any specific setting. Further, extracted DNA from parasite-contaminated soil could be used for surveillance of genetic resistance markers for anthelmintic and antiprotozoal drugs.

## 5. Soil and Water Assays across Field Sites in Latin America

Other research groups have developed methods for molecular testing of soil and other samples for STH parasites [20]. Our soil sampling method has the advantage of easy use and standardization across laboratories. The methods can be easily adapted for other environmental samples, such as household dust. Additionally, it offers the flexibility of a simple extraction method that can be performed in unsophisticated laboratories at field sites using inexpensive and widely available equipment with more centralized molecular testing where appropriate (Figure 1). The assays are currently being standardized across eight collaborating laboratories within a Latin American research network. Our sampling method draws on standardized multi-parallel qPCR assays developed for molecular diagnostics of enteric parasites in stool samples in resource-limited settings [7].

Further, the technique allows relatively high throughput testing of samples when using a 96-well plate format. In contrast, the high specificity of the assays allows human and animal parasites to be distinguished easily. Further studies must define the number of soil samples required to estimate potential transmission risks (or exclude significant risks) for a specific infection in a community or defined geographic area.

## 6. The Assays Have Wide Potential Applicability

The use of molecular assays to detect enteric parasites in soil and other environmental samples has a wide variety of potential applications: examples of potential uses are provided in Table 1. For example, in the case of STH control programs, mapping of prevalent STH in unmapped regions could use soil sampling as a rapid method before stool surveys to determine the need for preventive chemotherapy in communities. The technique may be particularly useful in populations of low parasite transmission where infections are likely undetectable by standard microscopic methods. Given the growing interest in moving beyond morbidity control to elimination of transmission of STH infections in some formerly endemic settings [21], in populations where STH transmission is thought to have been interrupted or is close to being interrupted, soil sampling may be more sensitive than screening of stools at a populational level. Longitudinal sampling could be performed in sentinel communities, as with other helminth elimination programs [22], with sampling focused on collecting relatively few soil samples from community centers or schools where ongoing or recrudescence of transmission could be monitored. In such circumstances, viability measurement will be important as intact DNA in old and degenerate eggs may otherwise give positive results. Similarly, post-treatment and post-certification surveillance could be carried out most efficiently using soil sampling at key sites in sentinel communities. It may be important to do surveillance for the emergence of genetic markers of resistance to benzimidazole drugs under conditions of periodic preventive chemotherapy, particularly if community-wide mass drug treatments are being given [23].

Genetic markers of resistance could be detected in soil samples before a loss of drug efficacy is detectable in treated populations, and community soil sampling would allow such surveillance to be performed relatively easily and cost-effectively. Control programs for other enteric parasites, if and when considered feasible and cost-effective, could use a similar strategy for initial mapping followed by measurement of impact on the transmission of Water, Sanitation, and Hygiene (WASH) strategies or chemotherapy.

## 7. Conclusions

The availability of molecular assays for the detection of enteric parasites in soil and other environmental samples using high throughput methodology that can be applied easily in resource-limited settings provides a potentially valuable tool for the surveillance of transmission risk in endemic populations, as well as for the monitoring of the emergence of drug resistance. Although further field evaluation of these methods will be required in various settings, these methods could complement stool collections for many research and surveillance uses, particularly in populations with limited acceptability and where repeated sampling may be required.

## Figures and Tables

**Table 1:** Examples of potential uses of soil and other environmental sampling for detecting enteric parasites in the environment. STH—soil-transmitted helminths. WASH—water, sanitation, and hygiene.

Potential Application	Sample	Indication	Sampling Sites	Parasites
STH control/elimination programs	Soil	Initial community mapping; monitoring for transmission interruption; post-treatment and post-certification surveillance; surveillance for drug resistance markers. Question: can environmental parameters be used to define transmission ‘break-points’ and will these breakpoints differ for distinct STH parasites?	Community centers/schools; households: transmission monitoring could be carried out in sentinel communities (either in community centers or in high-transmission households)	STH
Control programs for other enteric parasites/WASH strategies	Soil	Surveillance for key enteric parasites; surveillance for emergence of genetic resistance markers to common antiparasitic drugs	Community centers, schools, and households	STH and pathogenic protozoa.
Environmental monitoring	Soil	Animal husbandry	Farms and animal pens	STH
Clinical and epidemiological studies	Soil	Detection of low-level transmission where changes in prevalence may affect clinical disease (e.g., *A. lumbricoides* and respiratory disease; *T. trichiura* and rectal prolapse; etc.)	Households or schools	STH
Epidemiological studies	Soil	Detection of environmental contamination with zoonotic infections; One Health	Households; play areas; parks; schools; community centres; etc.	*Toxocara* spp.; *G. intestinalis* and *Cryptosporidium* spp.); zoonotic hookworms
Intervention studies	Soil	Measuring the impact of WASH or environmental decontamination to kill parasite stages in the environment using chemicals or biological agents; One Health interventions	Households; schools; communal areas	A wide variety of parasites, including zoonotic infections
Environmental monitoring	Wastewater/sludge/water sources/food sources	Outbreak investigations in non-endemic settings; analysis of sludge, biosolids, and compost as fertilizers or soil conditioners, etc; analysis of food/vegetables	Reservoirs, sewage plants, fresh water, market food	Pathogenic protozoa (i.e., *G. intestinalis* and *Cryptosporidium* spp.) and STH
Epidemiological studies	Soil/dust/water	Detection of sources of transmission of enteric and non-enteric parasites in household samples	Different areas outside (porch/play area, front door, latrine, etc.) and inside the house (entrance, bed, food preparation area, etc.)	A wide variety of parasites with an environmental reservoir
Climate change	Soil/dust/water	Longitudinal detection of parasites around and inside built environments to determine impact of climate change on parasite contamination	Households; schools; communal areas	A wide variety of parasites with an environmental reservoir
